# Cigarette-Smoking Intensity and Interferon-Gamma Release Assay Conversion among Persons Who Inject Drugs: A Cohort Study

**DOI:** 10.1155/2012/828106

**Published:** 2012-12-10

**Authors:** Sanghyuk S. Shin, Manuel Gallardo, Remedios Lozada, Daniela Abramovitz, Jose Luis Burgos, Rafael Laniado-Laborin, Timothy C. Rodwell, Thomas E. Novotny, Steffanie A. Strathdee, Richard S. Garfein

**Affiliations:** ^1^San Diego State University, 5500 Campanile Drive, San Diego, CA 92182-4162, USA; ^2^Division of Global Public Health, Department of Medicine, School of Medicine, University of California San Diego, 9500 Gilman Drive, MC-0507, San Diego, CA 92093-0507, USA; ^3^Patronato Pro-COMUSIDA, Ninos Heroes No. 697, Zona Centro, Tijuana, BC, Mexico; ^4^Parque Industrial Internacional, Universidad Autonoma de Baja California, Calzada Universidad 14418, Tijuana, BC, Mexico

## Abstract

We analyzed data from a longitudinal cohort study of persons who inject drugs (PWID) in Tijuana, Mexico, to explore whether cigarette smoking increases the risk of interferon gamma release assay (IGRA) conversion. PWID were recruited using respondent driven sampling (RDS). QuantiFERON-TB Gold In-Tube (QFT) assay conversion was defined as interferon-gamma concentrations <0.35 IU/mL at baseline and ≥0.7 IU/mL at 18 months. We used multivariable Poisson regression adjusted for RDS weights to estimate risk ratios (RRs). Of 129 eligible participants, 125 (96.9%) smoked at least one cigarette during followup with a median of 11 cigarettes smoked daily, and 52 (40.3%) had QFT conversion. In bivariate analysis, QFT conversion was not associated with the number of cigarettes smoked daily (*P* = 0.716). Controlling for age, gender, education, and alcohol use, the RRs of QFT conversion for smoking 6–10, 11–15, and ≥16 cigarettes daily compared to smoking 0–5 cigarettes daily were 0.9 (95% confidence interval (CI), 0.5–1.6), 0.5 (95% CI, 0.3–1.2), and 0.7 (95% CI, 0.3–1.6), respectively. Although this study did not find an association between self-reported smoking intensity and QFT conversion, it was not powered sufficiently to negate such an association. Larger longitudinal studies are needed to fully explore this relationship.

## 1. Introduction

Evidence has accumulated over the years which demonstrates a causal relationship between tobacco use and increased tuberculosis (TB) morbidity and mortality [1–6]. However, the strength of evidence for this relationship varies by TB outcome [3]. For example, while high-quality longitudinal cohort studies provide strong evidence that tobacco use increases the risk of TB disease, the evidence for the relationship between tobacco use and the risk of *Mycobacterium tuberculosis* infection is relatively weak [3, 7, 8]. Previous studies exploring this relationship utilized cross-sectional or case-control methodologies to determine the association between “ever” or “current” smoking and lifetime infection with *M. tuberculosis* as determined by a single tuberculin skin test (TST) result [9–14]. Therefore, these studies were not able to assess the temporality between tobacco use and *M. tuberculosis *infection. For example, a participant infected with *M. tuberculosis *as a child who subsequently began smoking years later would contribute to the positive association between smoking and TST positivity. 

An improved understanding of the relationship between cigarette smoking and *M. tuberculosis *infection would help inform the implementation of tobacco control efforts as a part of global TB interventions. However, due to the low incidence of *M. tuberculosis* infection in most populations, conducting longitudinal cohort studies to strengthen the evidence regarding this relationship would necessitate the enrollment and long-term followup of a large number of participants. Furthermore, while interferon gamma release assays (IGRAs) have been shown to have higher specificity than TSTs for the diagnosis of latent TB infection (LTBI), no study has explored the effect of tobacco use on serial IGRA test results [15, 16].

The objective of the present study was to investigate the association between level of cigarette smoking and IGRA conversion among persons who inject drugs (PWID) in Tijuana, Mexico, a population at high risk for *M. tuberculosis *infection. Previous studies using this cohort showed a high baseline LTBI prevalence LTBI of 67% and an 18-month IGRA conversion rate of 28.7% to 51.9%, depending on the definition of conversion used [17, 18]. We hypothesized that higher levels of cigarette smoking in this population would be associated with increased risk for IGRA conversion in a dose-response relationship.

## 2. Materials and Methods

### 2.1. Study Design and Population

We analyzed data from a longitudinal cohort study of PWID in Tijuana, Mexico that sought to determine risk factors for incident HIV, TB, and syphilis [19]. Study recruitment and data collection methods have been described in detail previously [19]. Briefly, eligible study participants were ages 18 years or older, had injected illicit drugs within the previous month, and had no plans to move from Tijuana during the followup period. Participants were recruited through respondent-driven sampling (RDS), which relies on recruiting participants through referrals from previously enrolled participants [20, 21]. RDS allows for the derivation of population-representative estimates of prevalence and risk factors by adjusting for the information collected on the participants' social networks during analysis [20, 21]. Enrolled participants made study visits at baseline and at 6, 12, and 18 months. To increase retention, community outreach workers actively contacted participants to remind them of their followup appointments. Participants were also provided with $10 at baseline and $5 at followup visits as compensation for their time and travel expenses. Only participants who tested IGRA negative at baseline and who had IGRA results available at 18 months were included in the present analysis. Institutional Review Boards at University of California, San Diego and the Tijuana General Hospital reviewed and approved the study protocol, and informed consent was obtained from all participants.

### 2.2. Measures

An in-depth questionnaire was administered via person-to-person interview at each visit. The questionnaire contained items on demographic characteristics and substance use behavior, including injection and noninjection use of illicit drugs, alcohol consumption, and cigarette smoking. Cigarette smoking was ascertained by first asking, “Have you smoked cigarettes in the past 6 months?” Participants who responded “Yes” were asked, “In the past 6 months, how many cigarettes did you usually smoke per day?” Based on preliminary analysis, we anticipated a high prevalence of cigarette smoking in this population and, consequently, insufficient number of nonsmokers for categorization. Therefore, we used the average number of cigarettes smoked daily during the 18-month study period as the exposure of interest. This exposure was stratified into quartiles (0–5, 6–10, 11–15, and ≥16 cigarettes) for the primary analysis.

 IGRA conversion at 18 months was ascertained using QuantiFERON-TB Gold In-Tube ((QFT) Cellestis, VIC, Australia). For this test, whole blood samples were collected in three separate tubes: a Nil Control tube, TB Antigen tube, and a Mitogen Control tube. The tubes were incubated at 37°C for 16 to 24 hours and centrifuged. The interferon-gamma (IFN-*γ*) released in the Nil Control tube was then measured using enzyme-linked immunosorbent assay (ELISA) and subtracted from that found in the TB Antigen Tube. QFT was administered at baseline for all participants. However, because of an unexpected delay in procuring the supplies necessary for specimen collection and testing, only a subset of the participants who were QFT negative using the manufacturer recommended cutoff of <0.35 IU/mL at baseline were retested at 18 months. For the primary analysis, we used a previously published conservative definition of QFT conversion (i.e., baseline IFN-*γ* < 0.35 IU/mL and IFN-*γ* ≥ 0.70 IU/mL at followup), which reduces false positive conversions that could potentially arise due to within-subject variability observed in serial QFT tests [22]. In a secondary sensitivity analysis, we used the cutoff of 0.35 IU/mL at 18 months to define conversion. 

### 2.3. Statistical Analysis

The Pearson's *χ* ^2^ test was used for comparisons involving categorical variables, and the Wilcoxon rank-sum and the Kruskal-Wallis tests were used for continuous variables. We considered statistical tests to be significant at *α* of 0.05. We constructed Poisson regression models with robust variance estimation, via generalized estimating equation (GEE), to determine risk ratios (RRs) for QFT conversion for participants in each smoking exposure quartile compared to those in the first quartile [23, 24]. The models were weighted by inverse probability weights derived using the RDS Analytical Tool [25]. The GEE algorithm also accounted for clustering by recruiter assuming an exchangeable correlation structure.

The base model included covariates representing established risk factors for *M. tuberculosis* infection, including age, gender, education, and alcohol use, regardless of their association with QFT conversion in our study population. We also evaluated the effect of drug use behavior using the “change-in-estimate” approach; drug use variables were added to the base model only if their inclusion changed the RRs between smoking and QFT by >10% [26]. Drug use variables evaluated included frequency and duration of heroin, methamphetamine, cocaine and marijuana use, including smoking of these substances. To account for the possible loss of statistical power due to overfitting the final model with covariates, we also constructed a reduced model that included the stratified smoking exposure variable and only the covariates that were statistically significant predictors of QFT conversion. For the final model, we calculated tolerance and condition index statistics to assess multicollinearity, and Pearson residuals, Cook's distance, and leverage statistics to identify outlier observations [27]. SAS 9.3 (Cary, North Carolina) was used for all analyses.

## 3. Results

 Of the 1056 participants enrolled during April 2006–April 2007, 341 had negative QFT (IFN-*γ* <0.35 IU/mL) at baseline. Of these, 129 (37.8%) who had QFT results available at 18 months were included in the analysis. Among included participants, the median age was 38 (interquartile range (IQR) = 32–43), 107 (82.9%) were male, and 99 (76.7%) had obtained middle school education or less (Table 1). Nearly all of the included participants (96.9%) reported smoking at least one cigarette during the followup period. On average, participants included in the analysis smoked fewer cigarettes per day compared to the 212 participants who were excluded due to missing QFT results at 18 months (median of 10.5 [IQR = 6–15] versus 12.5 [IQR = 7–19] cigarettes per day, resp., *P* = 0.023). None of the other characteristics differed between included and excluded participants (Table 1). At 18 months, 52 (40.3%) participants met the primary QFT conversion definition.

**Figure 1:**
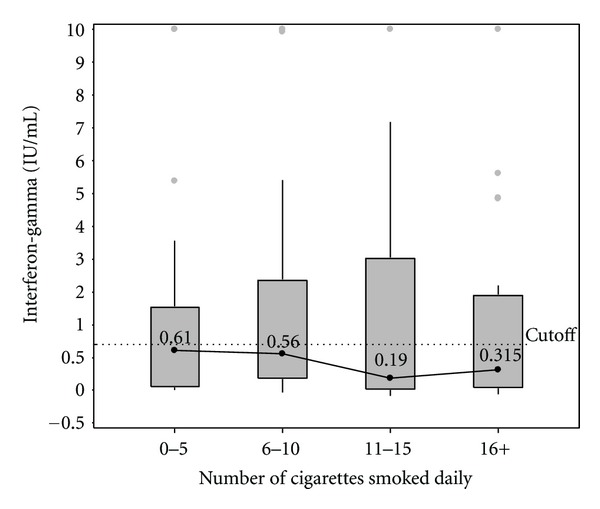
Median IFN-*γ* and interquartile range at 18 months by quartiles of number of cigarettes smoked. IFN-*γ* > 10 IU/mL were set to 10 IU/mL due to imprecision at high concentration levels. The dotted line represents the 0.70 IU/mL cutoff which was used to define QFT conversion. IFN-*γ*: interferon-gamma. QFT: QuantiFERON-TB Gold In-Tube.

Across quartiles of self-reported daily cigarettes smoked, the median IFN-*γ* concentrations were 0.61, 0.56, 0.19, and 0.315 IU/mL, respectively (Figure 1), and the proportion of participants with QFT conversion was 43.4%, 44.4%, 40.5%, and 30.8%, respectively (Table 2). There was no association between IFN-*γ* distribution or QFT conversion across quartile levels of daily cigarettes smoked (*P* = 0.523 and *P* = 0.716, resp.). In the bivariate model adjusted for RDS weights, which included only the smoking quartiles as the independent variable, the RRs for QFT conversion for each quartile of daily number of cigarettes smoked compared to the lowest exposure quartile were 0.75 (95% confidence interval [CI] 0.37–1.55), 0.53 (95% CI 0.23–1.20), and 0.59 (95% CI 0.24–1.43), respectively (Table 3).

**Figure 2:**
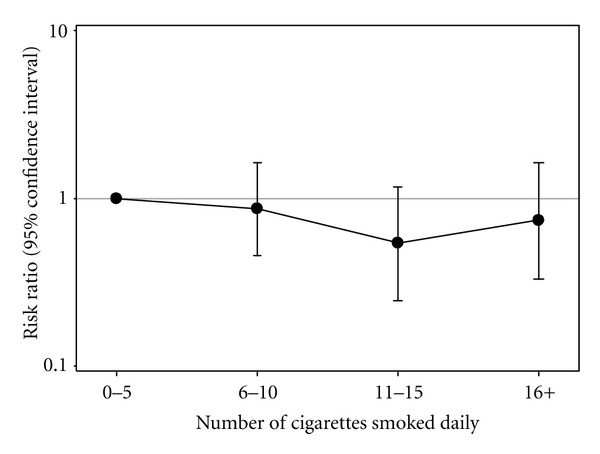
Adjusted risk ratios for QFT conversion (IFN-*γ* ≥ 0.70 IU/mL) at 18 months based on the final multivariable Poisson regression model with robust variance which included the following covariates: quartiles of the number of cigarettes smoked daily, education, age, gender, and alcohol use. IFN-*γ*: interferon-gamma. QFT: QuantiFERON-TB Gold In-Tube.

 In multivariable analysis adjusted for RDS, inclusion of drug use variables to the base model did not change the association between cigarette smoking quartiles and QFT conversion. Therefore, the final model consisted of daily cigarette smoking quartiles, age, gender, education, and alcohol use as independent variables (Table 3). The adjusted RRs for QFT conversion for each quartile of daily number of cigarettes smoked compared to the lowest exposure quartile were 0.86 (95% CI 0.46–1.63), 0.54 (95% CI 0.25–1.17), and 0.74 (95% CI 0.33–1.64), respectively (Table 3; Figure 2). There was no statistically significant difference in the risk of QFT conversion at any of the quartiles of daily cigarettes smoked compared with that of the lowest exposure quartile. Furthermore, age, gender, education, and alcohol use were not statistically significant predictors of QFT conversion. In the reduced model that included the smoking variables and educational attainment only, having attained less than high school education compared with higher education was found to increase the risk of QFT conversion (RR = 2.83; 95% CI 1.08–7.42). As with the full model, higher levels of daily cigarette smoking exposure quartiles were not associated with QFT conversion risk in this model (Table 3).

All tolerance estimates were greater than 0.10, and the highest condition index was 11.8 in the final model, indicating that multicollinearity did not affect our findings. We found five potential outliers based on residual and influence statistics, but removing these had no effect on our findings. Additionally, using a cutoff of 0.35 IU/mL instead of 0.70 IU/mL at 18 months to define conversion and fitting the final model with daily number of cigarettes smoked as a continuous variable did not alter our findings. In post hoc power analysis, assuming 43.3% QFT conversion risk that we found among participants in the lowest cigarette-smoking quartile, our sample size of 129 provided 28.1%, 55.6%, and 82.6% power to detect a RR of 1.4, 1.6, and 1.8, respectively, for QFT conversion among participants in the highest exposure quartile.

## 4. Discussion

In our analysis of longitudinal cohort data from PWID in Tijuana, we were not able to detect a dose-response relationship between the number of cigarettes smoked per day and QFT conversion. Previous studies evaluating dose-response relationships between cigarette-smoking and *M. tuberculosis *infection have shown mixed results for this putative association. A study of population survey data in South Africa found no evidence of a dose-response relationship between pack-years and TST positivity [9]. Likewise, a study of people with silicosis in Hong Kong found no relationship between the number of cigarettes smoked per day or cigarette pack-years and TST positivity [10]. In contrast, a study of prisoners in Pakistan found that TST prevalence increased with number of cigarettes smoked per day [11]. However, these previous studies employed cross-sectional or case-control study designs, which limit their ability to evaluate temporality between cigarette-smoking exposures and *M. tuberculosis* infection. 

Cigarette smoking has been hypothesized to increase the risk of *M. tuberculosis *infection by adversely affecting the innate immune system of the host and/or causing structural damage to the respiratory tract [28]. First, exposure to cigarette smoke might impair the ability of alveolar macrophages to clear the *M. tuberculosis* bacilli before T cells are primed for adaptive immunity. Under this model, increased exposure to cigarette smoke in the lungs would result in increased acute susceptibility to *M. tuberculosis* infection. We were unable to generate evidence to support this model in our study. Smoking also impairs the mucociliary clearance of pathogens and causes other changes to the respiratory tract that could increase the risk for *M. tuberculosis *infection over time [28–30]. Because we did not collect information regarding lifetime history of smoking, we were not able to evaluate the long-term effect of cigarette smoking on *M. tuberculosis *infection. 

Our findings should be interpreted with consideration of the following limitations. First, we were not able to compare QFT conversion risk between smokers and nonsmokers because nearly all of our study participants reported smoking during the study followup period. If even low levels of cigarette smoking increase IGRA conversion substantially, there might have been minimal increased risk for higher frequency smokers, and our study might not have had sufficient power to detect a dose-response relationship. While we had adequate sample size to detect a RR of 1.8 or greater for QFT conversion between participants in the lowest and highest smoking exposure quartiles, the study was under-powered to conclude that there is no association between smoking and QFT conversion. We were also unable to control for history of exposure to persons with TB disease, which is a necessary causal factor for incident *M. tuberculosis* infection. The inclusion of participants who were not exposed to persons with TB disease in our analysis could have biased our results towards the null. However, controlling for a proxy variable “Have you ever known someone who had TB?” did not alter our findings (data not shown). Future longitudinal studies should investigate the risk of cigarette smoking on *M. tuberculosis* infection among study participants recruited from persons with known history of exposure to someone with TB disease.

 It is also possible that our study participants were already at high risk for *M. tuberculosis *infection due to other risk factors, which might have overshadowed an incremental increase in risk due to cigarette smoking. In addition, as with TSTs, QFT assays have significant within-subject variability such that conversions and reversions often occur around the 0.35 IU/mL cutoff during serial testing even among persons who are at low risk for *M. tuberculosis *infection [31, 32]. While we used a conservative definition of QFT conversion to minimize misclassification in our analysis, the conversions observed in our study might not represent incident *M. tuberculosis *infection. Furthermore, since the recommended QFT cutoff of 0.35 IU/mL was derived to maximize specificity for *M. tuberculosis *infection, the use of this cutoff as an inclusion criterion could have resulted in the inclusion of some participants who were already infected with *M. tuberculosis* at baseline. However, restricting the analysis to only those participants with baseline QFT of <0.20 did not alter our findings (data not shown). Participants included in our study smoked fewer cigarettes than the participants who were excluded due to unavailable QFT results at 18 months. Therefore, our findings might not be generalizable to all PWID at risk for *M. tuberculosis* infection. Smoking levels were ascertained by self-report, which might have insufficient precision to evaluate a dose-response relationship. Lastly, we did not collect information regarding secondhand smoke exposure, which has been shown to be associated with *M. tuberculosis* infection among children [33].

## 5. Conclusions

The present study is the first longitudinal cohort study to explore the relationship between cigarette-smoking intensity and *M. tuberculosis *infection, and the first to use IGRA conversion as the outcome. Given our findings and the limitations of previous research on this topic, additional research is needed to determine whether there is a causal relationship between smoking and *M. tuberculosis* infection. For example, a recent mathematical modeling study concluded that intensified tobacco control efforts could prevent 27 million TB-related deaths by 2050 [34]. However, the authors of that study assumed a RR of 2.0 for the effect of smoking on *M. tuberculosis *infection in their model to arrive at this conclusion. Stronger evidence from larger longitudinal studies is needed to justify such assumptions. Ideally, such a study would be conducted among persons at high risk for *M. tuberculosis *infection, such as those with household exposure to persons with TB disease, and consists of sufficient numbers of smokers and nonsmokers. While the evidence of a causal relationship between smoking and *M. tuberculosis *infection is weak, substantial evidence exists that implicates smoking as an independent risk factor for the development of TB disease [1–8]. Therefore, integration of tobacco and TB control interventions remains a high priority for global health [1]. 

## Figures and Tables

**Table 1:** Demographic and behavioral characteristics of participants with negative QFT results (IFN-*γ* < 0.35 IU/mL) at baseline, included versus not included in the analysis; Tijuana, Mexico, 2006–2008.

Characteristic	Included	Not included	*P* value*
	*N* = 129	*N* = 212	
	*n* (%)	*n* (%)	
Gender			0.806
Male	107 (82.9)	178 (84.0)	
Female	22 (17.1)	34 (16.0)	
Age, median (IQR)	38 (32–43)	37 (30–42)	0.247
Education			0.389
Up to primary	39 (30.2)	76 (35.8)	
Primary to middle	60 (46.5)	83 (39.2)	
High school and higher	30 (23.3)	53 (25.0)	
Unstable housing			0.357
No	113 (87.6)	178 (84.0)	
Yes	16 (12.4)	34 (16.0)	
History of incarceration			0.719
No	78 (60.5)	124 (58.5)	
Yes	51 (39.5)	88 (41.5)	
HIV infection			0.366
No	123 (95.3)	197 (92.9)	
Yes	6 (4.7)	15 (7.1)	
Alcohol			0.154
None	76 (58.9)	134 (63.2)	
Less than twice per week	31 (24.0)	57 (26.9)	
Twice per week or more	22 (17.1)	21 (9.9)	
Smoked cigarette during study periods (18 months)			0.919
No	4 (3.1)	7 (3.3)	
Yes	125 (96.9)	205 (96.7)	
Number of cigarettes smoked daily, median (IQR)	10.5 (6–15)	12.5 (7–19)	0.023
Number of cigarettes smoked daily (quartiles)			0.058
0–5	30 (23.3)	41 (19.3)	
6–10	36 (27.9)	50 (23.6)	
11–15	37 (28.7)	49 (23.1)	
16+	26 (20.2)	72 (34.0)	
QFT conversion at 18 mos (IFN-*γ* ≥ 0.70 IU/mL)	
No	77 (59.7)	—	
Yes	52 (40.3)	—	

*P values for the difference between included versus excluded participants were generated using the Pearson's *χ* ^2^ test for categorical variables and the Wilcoxon rank sum for continuous variables.

IFN-*γ*: interferon-gamma; QFT: QuantiFERON-TB Gold In-Tube; IQR: interquartile range.

**Table 2:** QFT conversion (IFN-*γ* ≥ 0.70 IU/mL) at 18 months by quartiles of number of cigarettes smoked among persons who inject drugs in Tijuana, Mexico, 2006–2008.

Number of cigarettes smoked daily (quartiles)	QFT conversion	*P* value
0–5	13/30 (43.3)	0.716
6–10	16/36 (44.4)	
11–15	15/37 (40.5)	
16+	8/26 (30.8)	

*P value for the difference in QFT conversion at 18 months across quartiles was generated using the Pearson's *χ* ^2^ test.

IFN-*γ*: interferon-gamma; QFT: QuantiFERON-TB Gold In-Tube.

**Table 3:** Adjusted risk ratios for QFT conversion (IFN-*γ* ≥ 0.70 IU/mL) at 18 months based on multivariable Poisson regression models with robust variance; Tijuana, Mexico, 2006–2008.

Variable	Risk Ratio (95% Confidence Interval)		
	Bivariate Model	Reduced Model	Final Model
Number of cigarettes smoked daily (quartiles)			
0–5	1.00	1.00	1.00
6–10	0.75 (0.37–1.55)	1.04 (0.53–2.04)	0.86 (0.46–1.63)
11–15	0.53 (0.23–1.20)	0.73 (0.36–1.51)	0.54 (0.25–1.17)
≥16	0.59 (0.24–1.43)	0.79 (0.34–1.83)	0.74 (0.33–1.64)
Education			
High school or higher		1.00	1.00
Less than high school		2.83 (1.08–7.42)	2.60 (0.96–7.03)
Age			
+10 years			1.25 (0.80–1.94)
Gender			
Male			1.00
Female			0.81 (0.34–1.93)
Alcohol use			
<2x per week			1.00
≥2x per week			1.04 (0.52–2.08)

IFN-*γ*: interferon-gamma; QFT: QuantiFERON-TB Gold In-Tube.
